# Prevalence of reduced visual acuity among school-aged children and adolescents in 6 districts of Changsha city: a population-based survey

**DOI:** 10.1186/s12886-020-01619-2

**Published:** 2020-08-26

**Authors:** Menglian Liao, Zehuai Cai, Muhammad Ahmad Khan, Wenjie Miao, Ding Lin, Qiongyan Tang

**Affiliations:** 1 Aier School of Ophthalmology, Central South University, Changsha, China; 2 Changsha Aier Eye Hospital, Changsha, China; 3 Hunan Provincial Clinical Research Center for Ocular Surface Diseases, Changsha, China

**Keywords:** Reduced visual acuity, Cloud platform, Epidemiology, Risk factors

## Abstract

**Background:**

To calculate and evaluate the prevalence of reduced uncorrected distant visual acuity (UCDVA) in primary, middle and high schools in 6 districts of Changsha, Hunan, China.

**Methods:**

A population-based retrospective study was conducted in 239 schools in 6 districts of Changsha. After routine eye examination to rule out diseases that can affect refraction, 250,980 eligible students from primary, middle and high schools were enrolled in the survey. Then the uncorrected distant and near visual acuity of each eye were measured. Categories of schools, districts, grades, eye exercises and sports time were also documented and analyzed.

**Results:**

The overall prevalence of reduced UCDVA was 51.8% (95% confidence interval [CI]: 51.6–52.0%) in 6 districts of Changsha. Results of individual districts were as follows: Furong district 59.9%(95% CI: 57.9–61.8%), Tianxin district 62.3%(95% CI: 60.5–64.0%), Wangcheng district 47.8%(95% CI: 46.8–48.8%), Kaifu district 58.5%(95% CI: 58.0–58.9%), Yuhua district 47.0%(95% CI: 46.7–47.4%) and Yuelu district 52.6%(95% CI: 52.3–52.9%). The proportion of normal VA is seen to decrease from primary grade 3. The proportion of mildly reduced UCDVA is higher in primary grade 1 and 2. The proportion of moderately reduced UCDVA remains similar during 12 grades. The proportion of severely reduced UCDVA increases with grades. Multivariate analysis shows that the prevalence of reduced UCDVA is higher in key schools (risk ratio [RR] = 1.47, 95% CI 1.44–1.50) than non-key schools.

**Conclusions:**

According to the existing data analysis results, the prevalence of reduced UCDVA among primary, middle and high school students in Changsha is very high. Some effective measures need to be taken to prevent it.

## Background

According to existing surveys, reduced visual acuity (VA) has become the focus of public health in China [1]. The main cause of reduced VA is uncorrected refractive errors (URE) [2] that are mainly manifested as visual impairment (VI). According to the World Health Organization (WHO) survey, URE comprised 216.6 million of the global population in 2015 [3]. It can lead to a decline in learning ability and may compromise the individual’s academic performance, social communication, psychological development and quality of life [2, 4, 5]. Myopia is the commonest cause of VI in children and young adults, with a prevalence of 28.3% in the world [6]. It is reported that myopia affects 80–90% of teenagers in East Asia [7]. Meta-analysis showed that 69% of people from the age of 1 to 18 are myopic [8]. A survey carried out by researchers in southern China shows the prevalence of myopia in 13-year-olds and 17-year-olds as 36.8 and 53.9% respectively [9].

This study will analyze data collected from school children in Changsha city in order to assess the prevalence of reduced uncorrected distance visual acuity (UCDVA). Given the current critical state of visual health in China, we can suggest measures to prevent, control and treat reduced UCDVA of the students in their critical visual development stage.

## Methods

### Study population

This project is a population-based, retrospective study of students of 239 out of 445 schools in all 6 districts of Changsha from 2017 to 2018. It covers 4 out of 37 schools in Furong district, 4 out of 58 in Tianxin district, 10 out of 102 in Wangcheng district, and 65/65, 69/96 and 87/87 in Kaifu, Yuelu and Yuhua district respectively. Objects of investigation were in range from primary school grade 1(6–7 years old candidates) to high school grade 3 (17–18 years old candidates), 12 grades in total.

Ethical committee approval was sought from the Central South University and Changsha Aier Eye Hospital Review Board. The study was conducted adhering to the declaration of Helsinki involving human participants and the approved guidelines. Due to large work of written consent from such sample size, verbal consents have been approved by Central South University and Changsha Aier Eye Hospital Review Board, also students and their parents. After introducing examination procedures to students, parents, guardians and school committees, the vision data with informed consents will be uploaded to cloud platform. Finally, the survey will be carried out accompanied by the school staff after their approval.

### Eye examination

Students were first examined by experienced ophthalmologists from Changsha Aier Eye Hospital using portable slit lamp (YZ3; 66vision Ltd., Suzhou, China) to rule out any disorders of anterior segment that may affect refraction, such as glaucoma, lens opacity or other diseases [10, 11]. Students with pathological ocular conditions were excluded. Eligible participants then underwent measurement of uncorrected distance visual acuity (UCDVA) at 5 m (Standard Logarithmic Visual Acuity E chart) and uncorrected near visual acuity (UCNVA) at 40 cm uniocularly by technicians. UCDVA was reported in LogMAR form [12], UCNVA written from J1 to J7 represents the worsening of VA. UCDVA and UCNVA of all eyes were uploaded to the cloud platform and processed for data analysis. Participants without anterior segment abnormalities but with extremely poor VA were sent to hospital for further ophthalmic examination.

### Operational definition of reduced UCDVA

Vision worse than 0.00 was defined as reduced UCDVA, which was further divided into 3 groups according to the severity: mild: 0.000 to 0.175, moderate: 0.200 to 0.400, and severe: worse than 0.425.

### Questionnaire

Questionnaires were filled in by school faculties with simple items: 1. Is your school a key school or non-key school? 2. Are eye exercises carried out in your school? If so, how frequent are they? 3. Do you have any physical education classes in your school? If so, how often are they?

### Statistical analysis

Statistical Package for the Social Sciences (SPSS) 22.0 statistical software was used for data analysis. The prevalence of reduced UCDVA was defined as the proportion of students presenting with UCDVA worse than 0.00 in worse eye. Not only did we analyze the overall prevalence of the sample, we also evaluated the prevalence of reduced UCDVA by districts and grades respectively. Considering the counterbalance of participants distribution, a SNK-q test was run to analyze the prevalence difference among districts and grades. After homogeneity of variance test, types of schools were estimated in risk ratio (RR) and 95% confidence interval (CI) was determined based on one-way ANOVA test.

## Results

250,980 students were finally included in this investigation. Table 1 shows the distribution of participants by districts and grades. In this table, blank marks (“/”) represent missing data in Furong, Tianxin and Wangcheng districts due to tough schedule and thus unavailability of students who were preparing for entrance exams for further education, especially in secondary grade 3 and high grade 3. For this reason, 7195 (2.87%) subjects from secondary grade 3 and high grade 3 participated in this screening program.

**Table 1:** Distribution of participants. “/”: missing data

	Furong		Tianxin		Wangcheng		Kaifu		Yuhua		Yuelu		Total
	n	%	n	%	n	%	n	%	n	%	n	%	
Primary Grade 1	92	3.83%	134	4.41%	1905	19.54%	70.84	15.15%	12,937	15.36%	16,302	15.54%	38,454
Primary Grade 2	71	2.96%	86	2.83%	1985	20.36%	6720	14.39%	12,083	14.35%	14,497	13.82%	35,442
Primary Grade 3	94	3.92%	139	4.58%	1497	15.36%	6151	13.17%	11,285	13.41%	12,873	12.27%	32,039
Primary Grade 4	92	3.83%	85	2.81%	933	9.57%	5390	11.54%	10,746	12.77%	11,844	11.29%	29,090
Primary Grade 5	113	4.71%	157	5.17%	1084	11.12%	5586	11.96%	9704	11.53%	11,310	10.78%	27,954
Primary Grade 6	109	4.54%	72	2.37%	1060	10.87%	4923	10.54%	7835	9.31%	10,030	9.56%	24,029
Secondary Grade 1	758	31.56%	1101	36.25%	704	7.22%	4301	9.21%	7041	8.37%	9302	8.87%	23,207
Secondary Grade 2	564	23.49%	872	28.71%	581	5.96%	3841	8.22%	5466	6.49%	8218	7.83%	19,542
Secondary Grade 3	/	/	/	/	/	/	1858	3.98%	798	0.95%	3562	3.40%	6218
High Grade 1	508	21.16%	198	6.52%	/	/	514	1.10%	3577	4.25%	3112	2.97%	7909
High Grade 2	/	/	193	6.35%	/	/	229	0.49%	2699	3.21%	2998	2.86%	6119
High Grade 3	/	/	/	/	/	/	116	0.25%	/	/	861	0.82%	977
Total	2401	100%	3037	100%	9749	100%	46,713	100%	84,171	100%	104,909	100%	250,980

**Fig. 1:**
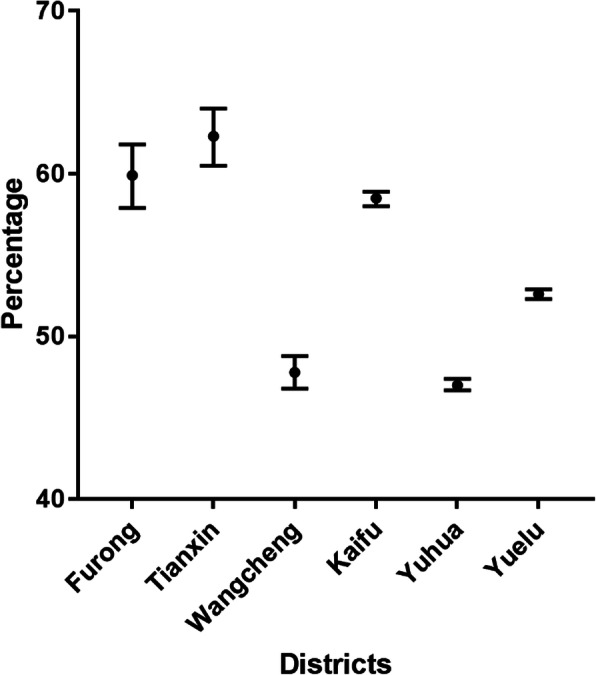
The prevalence of reduced uncorrected distance visual acuity in 6 districts

After analyzing the data, we found out that the overall prevalence of reduced UCDVA was 51.8% (95% CI 51.6–52.0%) in 6 districts of Changsha city. 3496(1.39%) students manifested severe unilateral visual impairment in both distance and near VA with the other eye’s VA being normal. Besides that, all UCNVA is normal with J1. Figure 1 and Table 2 show the prevalence of reduced UCDVA in each district. There was a statistically significant difference in the prevalence between districts (*P* < 0.05).

**Table 2:** The prevalence of reduced UCDVA in 6 districts

Districts	prevalence	95% CI
Furong	59.9%	57.9–61.8%
Tianxin	62.3%	60.5–64.0%
Wangcheng	47.8%	46.8–48.8%
Kaifu	58.5%	58.0–58.9%
Yuhua	47.0%	46.7–47.4%
Yuelu	52.6%	52.3–52.9%

**Fig. 2:**
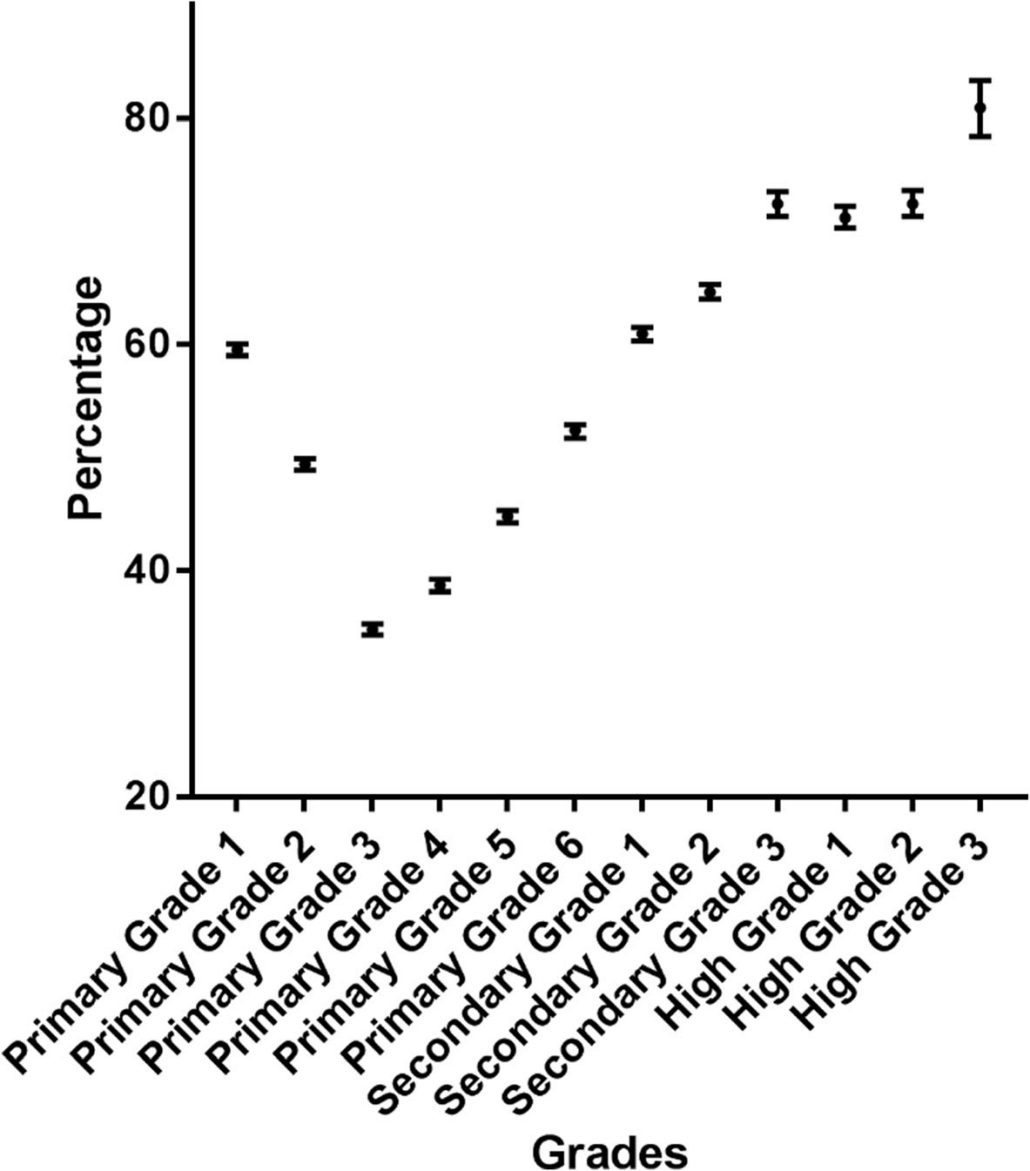
The prevalence of reduced uncorrected distance visual acuity in 12 grades

We also estimate the prevalence of reduced UCDVA in groups by grades as Fig. 2 and Table 3 show. There was a statistically significant difference in the prevalence between grades (*P* < 0.05).

**Table 3:** The prevalence of reduced UCDVA in 12 grades

Grade	prevalence	95% CI
Primary Grade 1	59.5%	59.0–60.0%
Primary Grade 2	49.4%	48.9–49.9%
Primary Grade 3	34.8%	34.3–35.3%
Primary Grade 4	38.7%	38.1–39.2%
Primary Grade 5	44.8%	44.2–45.3%
Primary Grade 6	52.4%	51.7–52.9%
Secondary Grade 1	60.9%	60.3–61.5%
Secondary Grade 2	64.6%	64.0–65.3%
Secondary Grade 3	72.4%	71.3–73.5%
High Grade 1	71.2%	70.3–72.2%
High Grade 2	72.4%	71.3–73.6%
High Grade 3	80.9%	78.4–83.3%

**Fig. 3:**
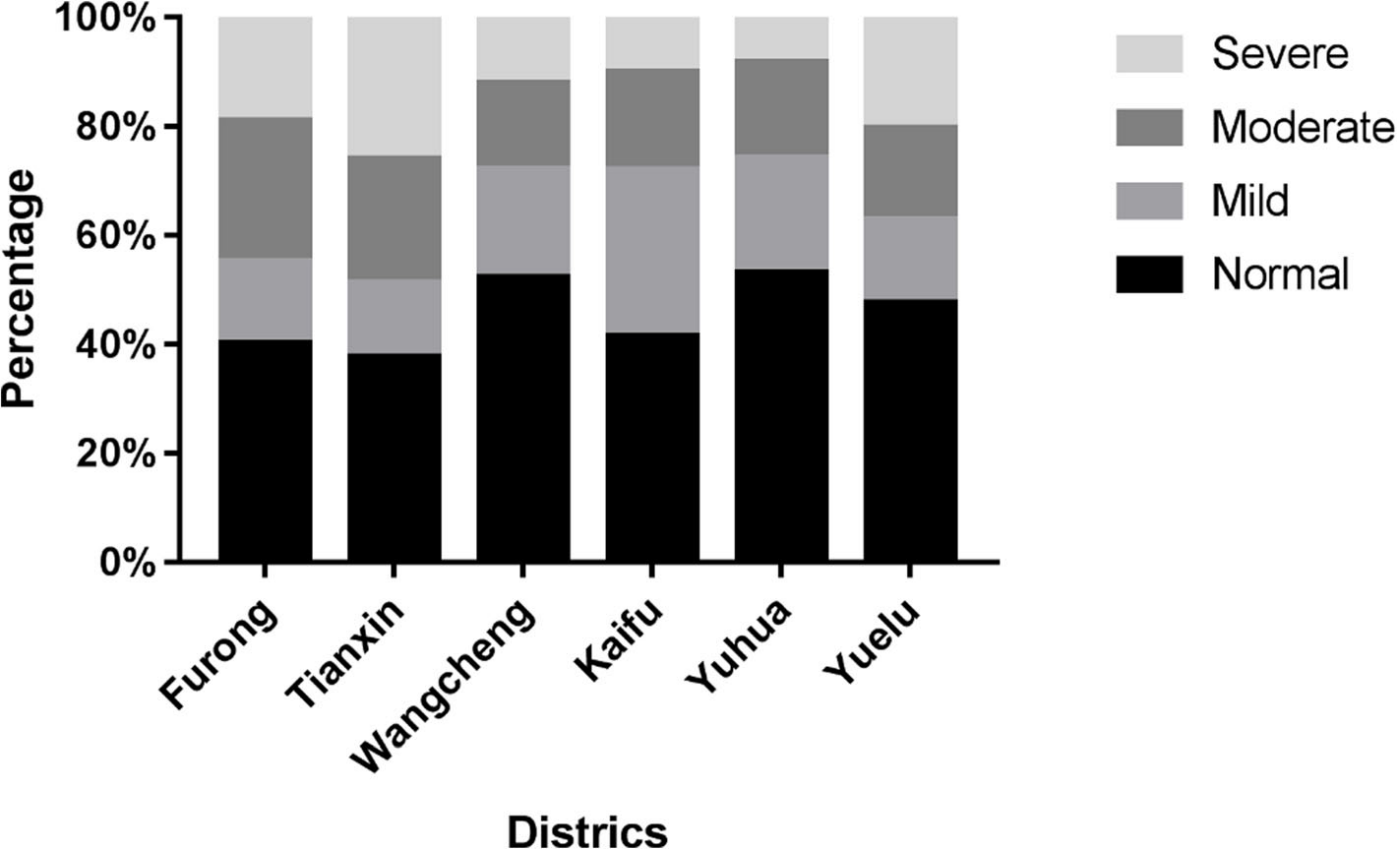
The distribution of reduced uncorrected distance visual acuity in 6 districts by intensity

**Fig. 4:**
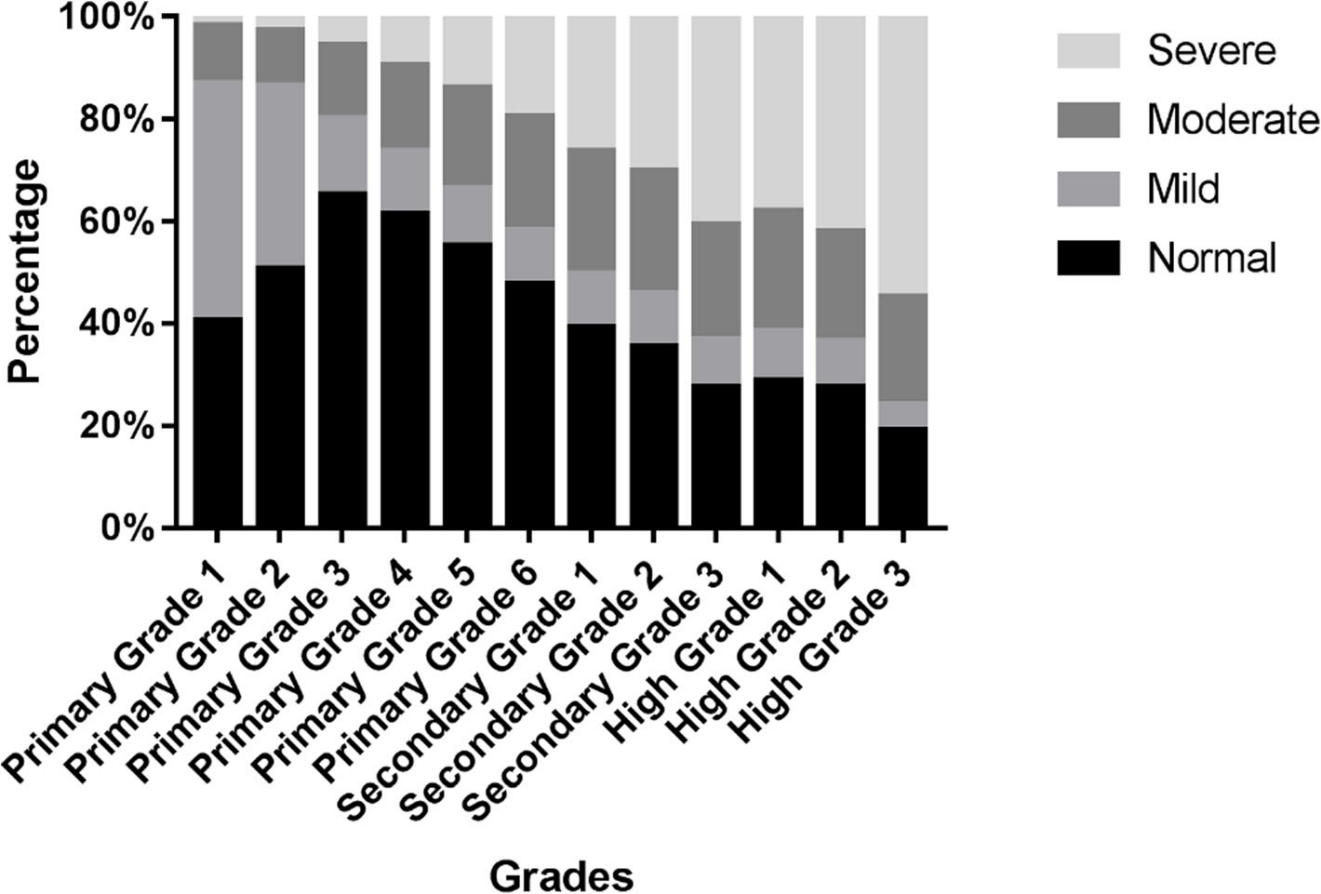
The distribution of reduced uncorrected distance visual acuity in 12 grades by intensity

Figure 3 and Fig. 4 presents the proportion of reduced VA by intensity of visual deficit in 6 districts and 12 grades respectively. Figure 3 shows similar levels of visual deficit in the 6 districts. Figure 4 shows that the proportion of intensity changes with grades. The detailed data is shown in the format of the percentage of normal VA/mildly reduced UCDVA/moderately reduced UCDVA/severely reduced UCDVA as shown in Table 4.

**Table 4:** Proportion of reduced UCDVA

Grade	Normal	Mildly	Moderate	Severely
Primary Grade 1	40.48%	46.40%	11.35%	1.77%
Primary Grade 2	50.59%	35.67%	10.86%	2.88%
Primary Grade 3	65.19%	14.75%	14.33%	5.73%
Primary Grade 4	61.33%	12.29%	16.78%	9.60%
Primary Grade 5	55.24%	11.06%	19.66%	14.04%
Primary Grade 6	47.61%	10.48%	22.21%	19.70%
Secondary Grade 1	39.09%	10.64%	23.95%	26.32%
Secondary Grade 2	35.35%	10.53%	24.01%	30.11%
Secondary Grade 3	27.63%	9.06%	22.72%	40.59%
High Grade 1	28.75%	9.62%	23.67%	37.96%
High Grade 2	27.57%	8.81%	21.64%	41.98%
High Grade 3	19.14%	5.02%	20.98%	54.86%

**Fig. 5:**
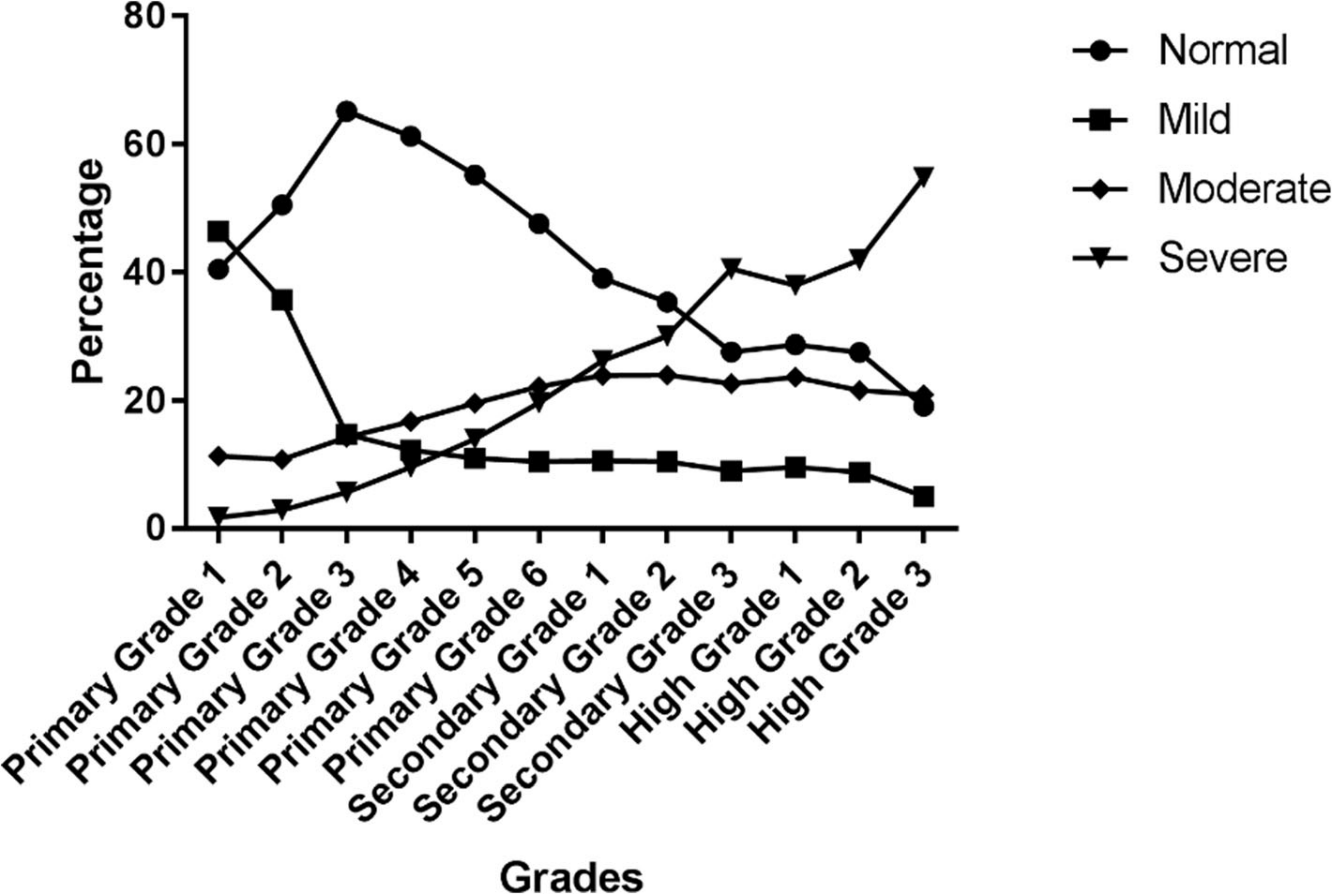
The tendency of prevalence of reduced uncorrected distance visual acuity by intensity in 12 grades

Figure 5 demonstrates the tendency with a curve. The proportion of normal VA can be seen to decrease from primary grade 3 onwards. The proportion of mildly reduced UCDVA is higher in primary grade 1 and 2. The proportion of moderately reduced UCDVA remains similar during 12 grades. The proportion of severely reduced UCDVA increases with grades.

Aiming to identify possible risk factors, homogeneity of variance test and one-way ANOVA test were run. We found out that the prevalence of reduced UCDVA was higher in key schools (RR = 1.47, 95% CI 1.44–1.50) than non-key schools. As all 239 schools maintained the same eye exercise and physical exercise period, no analysis was done between the reduced UCDVA and these two factors.

## Discussion

Located in the middle region of China, Changsha is the capital city of Hunan province with a population of 7.9 million. In order to estimate and evaluate the prevalence of reduced UCDVA in primary, middle and high school students of Changsha, Changsha Eye Health of Children and Adolescents Screening Program was initiated by Changsha Aier Eye Hospital with the agreement of local health government. In this program, 250,980 students from 239 schools in 6 districts of Changsha have been screened.

According to the statistical results, it is obvious that the prevalence of reduced UCDVA, 51.8%, is rather high in Changsha. In a previous study that investigated the prevalence of reduced UCDVA in ethnic Han students, similar results were seen, reporting a prevalence of reduced UCDVA of 66.6%, the major cause suspected being myopia [13]. In our study also, all participants except the 3496 students with severe unilateral visual impairment with reduced VA have a normal UCNVA (J1) yet an abnormal UCDVA, therefore myopia can be suspected as the main refractive error. Rise in the prevalence of myopia with age was confirmed by a study carried out in Qingdao city [14], which is in accordance with our findings since a statistically significant difference in prevalence was observed among grades. Another epidemiological study in 6 provinces of China reveals that the prevalence of myopia was 35.8% in age group 6–8 years old, 58.9% in 10–12 years old, 73.4% in 13–15 years old and 81.2% in 16–18 years old [15]. These age groups match with grades. For instance, students are about 6–12 years old in primary school, about 13–15 in secondary and 16–18 in high school. Our results are close to this study also. Statistical significance was observed for the prevalence of reduced UCDVA among districts which may be related to the different sample sizes in the groups.

Figures 4 and 5 show the significant increase in the proportion and prevalence of severely reduced UCDVA with increasing grade. This may be related to excessive near work as part of academic routine in Chinese education system. Excessively long study hours along with a sedentary lifestyle with less time spent outdoors and less exposure to sunlight increase the risk of myopia manifold [16, 17]. In Fig. 4 it is noticed that the proportion of mildly reduced UCDVA in primary grade 1 and primary grade 2 is high and this proportion gets lower with grade increase. Proportion of severely reduced VA however gets higher. This may attribute to ocular development during children’s hyperopic reserve stage, and this situation is consistent with other studies that revealed the predominant uncorrected refractive errors in children aged 6–7 years being hyperopia and astigmatism [18, 19]. However this was not furtherly examined in this study.

We analyzed suspected risk factors like categories of schools, eye exercises and sports time. Higher prevalence of reduced UCDVA in the key schools maybe be associated with more intensive educational burdens, longer hours of indoor lessons and longer study hours comparing with the non-key schools. The same finding was confirmed by an earlier study carried out in Beijing city [20]. The duration of eye exercises and physical exercise was the same in all 239 schools, in accordance with protocols implemented by Changsha Education Bureau. Therefore, no analysis could be done to interpret the influence of these two factors on VA.

We additionally found out that there were 3496 students having severe unilateral visual impairment in both distance and near VA. This may indicate anisometropia, unilateral amblyopia or retinal detachment, et al. Further examination and investigations are required in such cases to guide appropriate treatments.

The high prevalence of reduced UCDVA warns us about the deteriorating ocular health of children and adolescents and urges us to take appropriate measures. In the current scenario of insufficient eye care services, there are worldwide efforts to establish telemedicine and even artificial intelligence for eye care [21, 22]. For instance, UK has established the Hospital Eye Service (HES) with virtual technology, such as glaucoma monitoring service [23]. These technological achievements represent the direction of mainstream development of current medical technology. Under such circumstance, we conceive to set up a cloud platform to collect the visual data and analyze the prevalence of reduced UCDVA in screening program, to optimize human resource management and promote work efficiency, this can in line with these international developments.

The cloud platform includes a data logging system and a primary system. The students, guardians and school faculties are all allowed to log in with specified permission. We upload visual data after ocular examination and gather data for an overview. The data can be archived and downloaded whenever it is needed. Individual visual data file can be downloaded to clinic terminal when patients come to visit and be updated after the examination. Changes are then analyzed, and development trend predicted. Thus, we can offer appropriate refractive prescriptions and suggestions. If clients establish individual visual data file from childhood to adulthood, we are able to monitor their visual development over a long period of time. Even when these individuals age and come across age-related ocular diseases like age-related cataract, the additional information is valuable in offering refractive cataract surgery options [24]. This cloud platform also can be regarded as supplement for epidemiology data bank. Our privacy policy includes building firewall to raise data security. Besides data archive and push messages, we expect to explore more functions to run a better and more efficient cloud platform.

This study is the initial stage of our whole program. The observation indices were UCDVA and UCNVA only at this stage. More observation indices like corneal curvature, axial length, subjective refraction, autorefraction and others will be included in future. The refractive error and actual cause of reduced VA have not been determined at this time. Therefore it is only an assumption that the reduced uncorrected VA is due to myopia. 0.000 to 0.175 is normal vision for 6–7 years old children. This may represent an overestimation of reduced vision especially in the younger children in this study. Another limitation of this study is the different sample size among districts and grades. Further studies in minor sample size districts are expected to be conducted. More schools ought to be examined and appropriate schedules set to meet the available time of students in secondary grade 3 and high grade 3.

## Conclusion

Our study reveals a high prevalence of reduced UCDVA in school-aged children and adolescents in Changsha, which relates with grades, districts and key school categories, this may apply to further eye healthcare work, to instruct effective reduced UCDVA prevention and control. We going to deploy a cloud platform to archive vision data and provide data download and review to monitor visual development. Furthermore, with the assistance of the cloud platform, our work efficiency can be promoted dramatically.

## Data Availability

The datasets used and/or analyzed during the current study are available from the corresponding author on reasonable request.

## Abbreviations

VA: visual acuity
RR: risk ratio.
CI: confidence interval.
URE: uncorrected refractive error.
VI: visual impairment.
WHO: World Health Organization.
NHS: National Health Service.
HES: Hospital Eye Service.
UCDVA: uncorrected distance visual acuity.
UCNVA: uncorrected near visual acuity.
SPSS: Statistical Package for the Social Sciences.
